# The contribution of serum cortisone and glucocorticoid metabolites to detrimental bone health in patients receiving hydrocortisone therapy

**DOI:** 10.1186/s12902-020-00633-1

**Published:** 2020-10-10

**Authors:** Rosemary Dineen, Lucy-Ann Behan, Grainne Kelleher, Mark J. Hannon, Jennifer J. Brady, Bairbre Rogers, Brian G. Keevil, William Tormey, Diarmuid Smith, Christopher J. Thompson, Malachi J. McKenna, Wiebke Arlt, Paul M. Stewart, Amar Agha, Mark Sherlock

**Affiliations:** 1 Department of Endocrinology, Tallaght University Hospital, Dublin, Ireland; 2 Academic Department of Endocrinology, Beaumont Hospital and Royal College of Surgeons in Ireland, Dublin, Ireland; 3 Department of Chemical Pathology, Beaumont Hospital, Dublin, Ireland; 4 Metabolism Laboratory, St Vincent’s University Hospital, Dublin, Ireland; 5 School of Medicine and Medical Science, University College Dublin, Dublin, Ireland; 6 Manchester Academic Health Science Centre, University Hospital of South Manchester, The University of Manchester, Manchester, UK; 7 Biochemistry Department, University Hospital of South Manchester, Manchester, UK; 8 Institute of Metabolism and Systems Research, University of Birmingham, Birmingham, UK; 9 Centre for Endocrinology, Diabetes and Metabolism, Birmingham Health Partners, Birmingham, UK; 10 Medical School, University of Leeds, Leeds, UK

**Keywords:** Cortisone, Cortisol, Bone turnover markers, Hypopituitarism, Metabolites, Adrenal insufficiency

## Abstract

**Background:**

Glucocorticoid therapy is the most common cause of iatrogenic osteoporosis. Less is known regarding the effect of glucocorticoids when used as replacement therapy on bone remodelling in patients with adrenal insufficiency. Enhanced intracellular conversion of inactive cortisone to active cortisol, by 11 beta-hydroxysteroid dehydrogenase type 1(11β-HSD1) and other enzymes leading to alterations in glucocorticoid metabolism, may contribute to a deleterious effect on bone health in this patient group.

**Methods:**

Study design: An open crossover prospective study randomizing ten hypopituitary men, with severe ACTH deficiency, to three commonly used hydrocortisone dose regimens.

Measurements: Following 6 weeks of each regimen, patients underwent 24-h serum cortisol/cortisone sampling, measurement of bone turnover markers, and a 24-h urine collection for measurement of urinary steroid metabolites by gas chromatography-mass spectrometry (GC-MS). Serum cortisone and cortisol were analysed by liquid chromatography-mass spectrometry (LC-MS).

**Results:**

Dose-related and circadian variations in serum cortisone were seen to parallel those for cortisol, indicating conversion of ingested hydrocortisone to cortisone. The median area under the curve (AUC) of serum cortisone was significantly higher in patients on dose A (20 mg/10 mg) [670.5 (IQR 621–809.2)] compared to those on dose C (10 mg/5 mg) [562.8 (IQR 520.1–619.6), *p* = 0.01]. A negative correlation was observed between serum cortisone and bone formation markers, OC [1–49] (*r* = − 0.42, *p* = 0.03), and PINP (*r* = − 0.49, *p* = 0.01). There was a negative correlation between the AUC of night-time serum cortisone levels with the bone formation marker, OC [1–49] (*r* = − 0.41, *p* = 0.03) but there were no significant correlations between day-time serum cortisone or cortisol with bone turnover markers. There was a negative correlation between total urinary cortisol metabolites and the bone formation markers, PINP (*r* = − 0.39, *p* = 0.04), and OC [1–49] (*r* = − 0.35, *p* = 0.06).

**Conclusion:**

Serum cortisol and cortisone and total urinary corticosteroid metabolites are negatively associated with bone turnover markers in patients receiving replacement doses of hydrocortisone, with nocturnal glucocorticoid exposure having a potentially greater influence on bone turnover.

**Trial registration:**

Irish Medicines Board Clinical Trial Number – CT900/459/1 and EudraCT Number – 2007-005018-37. Registration date: 07-09-2007.

## Background

Glucocorticoids are widely used in the treatment of inflammatory, allergic, immunologic and malignant disorders. In adrenal insufficiency, glucocorticoids are given at doses intended to mimic the physiological concentrations and circadian rhythm of cortisol secretion [1]. Treatment of adrenal insufficiency consists of two or three daily oral doses of immediate-release hydrocortisone, which has a short half-life. As we and others have previously shown, this can result in serum cortisol peaks above and troughs below physiological levels [2–5]. Long-term over-replacement (even at relatively low exposure) of glucocorticoid therapy (as seen in iatrogenic Cushing’s syndrome) can cause weight gain, glucose intolerance and abnormal bone metabolism, leading to osteoporosis [6–8].

The deleterious effects of endogenous and exogenous glucocorticoid excess on bone health are well recognized. The risk of bone loss is greatest in the first few months following initiation of therapy, followed by a slower rate of loss with chronic use [9]. There is an increased risk of fractures associated with therapeutic immunosuppressive glucocorticoid therapy, and fractures occur at a higher bone mineral density (BMD) than that reported in postmenopausal osteoporosis [10].

Less literature exists on the effect of glucocorticoids used as replacement therapy for adrenal insufficiency on bone remodelling [11–14]. Some studies reported reduced BMD in all patients with primary adrenal insufficiency [12, 15] and other studies reporting this effect only in postmenopausal women receiving hydrocortisone replacement [16] or only in men [11, 17]. There is a paucity of data on the effect of glucocorticoid replacement on bone metabolism in patients with adrenocorticotropin (ACTH) deficiency/ secondary adrenal insufficiency. Peacey et al. demonstrated that a reduction in glucocorticoid dose by 30%, to 20 mg of hydrocortisone per day, was associated with a 19% increase in the bone formation marker osteocalcin (OC [1–49]) and a weak but significant negative correlation between absolute BMD and dose of hydrocortisone (HC) replacement [18]. Wichers et al. also demonstrated a significant increase in OC [1–49] as the dose of hydrocortisone decreased from 30 mg to 15 mg, however, there was no control group and no comment on the replacement status of the other pituitary hormones, which can have significant effects on bone health [19]. We have recently shown that there is an increase in OC [1–49] concentrations when the daily dose of hydrocortisone is decreased from 30 mg to 15 mg in a well-characterised cohort of hypopituitary patients on stable hormonal replacement therapy [20].

Several studies have shown, that in healthy controls, endogenous cortisol secretion is associated with BMD and the rate of bone loss. This has been assessed by serum cortisol measurement [21]*,* dynamic testing of the hypothalamic-pituitary-adrenal axis [22], salivary cortisol assessments [23, 24], and by urinary free cortisol [25]. Other authors have found the circadian rhythm of bone formation (but not of bone resorption) can be modified by changing cortisol circadian rhythm [26–28]*.*

In recent years, our knowledge of glucocorticoid action has expanded with the characterization of enzymes that regulate glucocorticoid action at the tissue level. The isoenzymes of the 11 beta-hydroxysteroid dehydrogenase system (11β-HSD) are responsible for intracellular glucocorticoid availability and are expressed in human synovial tissue and bone [29]. 11β-HSD type 2 converts the hormonally active cortisol (F) to inactive cortisone (E). In patients with ACTH deficiency, circulating cortisone is generated through 11β-HSD type 2 activity on ingested exogenous hydrocortisone therapy. In contrast, 11β-HSD type 1 converts the inactive glucocorticoid cortisone to active cortisol. 11β-HSD1 is expressed in human adult bone and cultured primary osteoblasts [30, 31]*.*

Enhanced intracellular conversion of cortisone to cortisol may contribute to a deleterious effect on bone mineral density, an assumption supported by the presence of polymorphisms within the HSD11B1 gene encoding 11β-HSD1 associated with low BMD and fracture risk in postmenopausal women without hypercortisolism [32]. Also, bone-specific responses to glucocorticoids have been shown to correlate with serum cortisone. Therefore, the presence of a tissue-specific conversion of inactive cortisone to active cortisol (i.e. 11β-HSD1) may be potentially biologically relevant [33].

On this background, our hypothesis was that circulating cortisone and tissue-specific metabolism of glucocorticoids impacts negatively on bone health in hypopituitary patients receiving hydrocortisone replacement therapy.

Our study aimed to examine in a prospective, cross-over randomized controlled manner in a group of male hypopituitary patients:

1. The daily cortisone and cortisol profile in patients receiving hydrocortisone therapy (previous studies have focused only on cortisol, not cortisone)
2. To assess the impact of different dosing regimens on bone turnover markers and compare this to healthy controls.
3. The association between serum cortisone and urinary measures of glucocorticoid metabolism with bone turnover markers.

## Methods

### Study patients

Ten adult male hypopituitary patients with known ACTH deficiency on dynamic testing were included in a randomized, controlled, crossover study of three different HC replacement regimens (results related to other aspects of this study have been published previously) [5, 20, 34]. Patients had been diagnosed and treated for pituitary tumours between 3 and 18 years before inclusion in the study.

The inclusion and exclusion criteria for study entry have been previously published [20]. Briefly, all patients were on stable appropriate pituitary hormone replacement, including growth hormone, without alteration in the dose for at least 3 months before and for the duration of the study. Hormone replacement therapy regimens were not adjusted during the study period, except for hydrocortisone dose, as per study protocol. Patients were matched for age, BMI and waist circumference with control subjects. No patient was taking calcium or vitamin D supplementation. Exclusion criteria included conditions associated with altered bone turnover such as Paget’s disease or known osteoporosis or fracture within the previous 1 year. We excluded patients on glucocorticoids for purposes other than ACTH deficiency and those on agents that interfere with corticosteroid or bone metabolism.

All patients were recruited through the pituitary clinic in Beaumont Hospital, Dublin, Ireland.

### Study design

The study design has been previously published [5, 20, 34, 35]. Subjects were randomized to a crossover protocol (in random order) of three commonly prescribed doses of HC; dose A – 20 mg 08.00 hours, 10 mg 16.00 hours; dose B – 10 mg 08.00 hours, 10 mg 16.00 hours and dose C – 10 mg 08.00 h, 5 mg 16.00  hours. These doses are frequently used in clinical practice [36]. At baseline and the end of each 6-week treatment schedule, patients were admitted, fasting, to the Clinical Research Facility at 07.30 h for 28 h. They underwent a physical examination that included blood pressure, weight, height, and waist circumference measurement. Following this examination, an 18 g cannula was placed in the antecubital fossa under aseptic technique. Basal samples were taken for cortisol, cortisol binding globulin (CBG), free T4, TSH, testosterone, gonadotropins, prolactin and insulin-like growth factor-I (IGF-I), parathyroid hormone (PTH), 25-hydroxyvitamin D (25 [OH] D), calcium, albumin and renal function. The cannula was then flushed with 10mls of a heparinised solution (heparin sodium, 100 units diluted in 100mls of 0.9% normal saline) to maintain patency for the full 28-h period. The use of diluted heparin in this manner does not affect any of the laboratory sampling techniques used subsequently [37] and since the first 5 ml of blood withdrawn at each time point was discarded there was little chance of contamination with such dilute levels of heparin. During this period the subjects had serum cortisol samples taken hourly through the indwelling cannula from the time of admission until midnight and were taken two-hourly from midnight until 0800 h the following morning.

Patients took the designated hydrocortisone dose that they had been taking for the preceding 6 weeks at 0800 h just after admission, at 1600 h and 0800 h the next morning. Meals were eaten at pre-defined times and lights were turned off at 23.00 h. The samples were allowed to stand at room temperature for 30 min to facilitate clotting before being centrifuged at 3000 rpm for 15 min, stored in 1 ml aliquots and frozen at − 20 and -80degrees centigrade for cortisol and CBG respectively until analysis.

The control participants for this study were ten healthy males, matched for age, BMI and waist circumference that underwent the same biochemical investigations and clinical examination but did not take exogenous cortisol. Additionally, patients and controls performed a 24-urine collection for measurement of urinary steroid metabolites.

Data regarding quality of life and serum cortisol profiles and the relationship with serum cortisol (but not cortisone or corticosteroid metabolites) and bone markers in this patient group have previously been published [5, 20, 34].

### Analytical methods

#### Bone markers and bone Remodelling

OC [1–49], CTX-I and PINP were measured using an electrochemiluminescence immunoassay on the Elecsys 2010 analyser (Roche Diagnostics, Mannheim, Germany) as previously described [20]. Bone ALP, a marker of both bone mineralisation and maturation was measured by an immunoenzymatic assay [20]. TRACP5b was measured by ELISA (Immunodiagnostic Systems Ltd., Boldon, UK) [20]. We calculated the PINP: CTX-I ratio as an approximation of bone remodelling balance [20].

#### Serum cortisone/ cortisol analysis by tandem mass spectrometry

Serum cortisol and cortisone were analysed by liquid chromatography-mass spectrometry following protein precipitation as previously described [38]. For cortisol, performance characteristics were as previously described [38]. For cortisone, inter-assay imprecision was 5.5, 3.9 and 3.8% at concentrations of 5.0, 50.0 and 150 nmol/L respectively. The limit of quantitation was determined to be 2.5 nmol/L and the assay was free from analytical interferences.

The area under the cortisone time curves in each patient (an estimate of the total circulating cortisone) was measured. We investigated the diurnal variation in circulating cortisone among the study population. Day-time serum cortisone was defined as the AUC of all serum samples taken from 08:00 until 19:00 inclusive. Night-time serum cortisone included all serum samples taken from 20:00 until 08:00 the following morning.

#### Urinary corticosteroid metabolite profiling by gas chromatography-mass spectrometry

Corticosteroid metabolites were analysed using urinary gas chromatography-mass spectrometry (GC-MS). GC-MS urinary steroid analysis was carried out in the Steroid Metabolome Analysis Core at the Institute of Metabolism and Systems Research, the University of Birmingham using previously reported methodology [39, 40]. Thirty-two steroids were targeted for selected-ion-monitoring analysis, including metabolites of androgens, mineralocorticoids and glucocorticoids (and their precursors).

The ratio of THF + 5α-THF/THE was used as a marker of 11β-HSD1 activity, providing the UFF/UFE ratio (reflecting 11β-HSD2 activity) was normal. Summation of THF + 5α-THF + THE + cortols + cortolones + UFF + UFE was used as a surrogate marker of 24-h total cortisol metabolites as previously validated [41].

#### Other biochemical indices

Serum 25OH-Vitamin D was measured by a competitive radioimmunoassay (Immunodiagnostic Systems Ltd., Boldon, UK) as previously described [20]. Serum PTH was measured using an electrochemiluminescent immunoassay on the Elecsys 2010 analyser (Roche Diagnostics, Mannheim, Germany) as described previously [20]. Renal function, albumin and calcium were measured using the Beckman Coulter AU5400 by standard laboratory protocols. Serum IGF-1, thyroid function, testosterone, prolactin concentrations were assessed using standard methodology as previously described [5].

### Statistical methods

Statistical analysis was performed using Prism for Windows version 5.0 (GraphPad Software, Inc., San Diego, CA, USA) software. Continuous data were summarized using means and S.D.s (or S.E.M.) if parametrically distributed or medians and inter-quartile ranges if non-parametrically distributed. Parametric data were compared using a paired *t*-test and non-parametric data were analysed using a Mann–Whitney test. Multiple comparisons were assessed using one-way ANOVA, with Kruskal–Wallis for non-parametric data. Repeated measures analysis was performed using the Friedman test and Dunn’s multiple comparison test. Associations between variables were analysed using Pearson correlation for parametric data and Spearman rank correlation for non-parametric data. We calculated that a sample of 7 patients per group was required for a power of 80% to detect a significant difference in bone turnover markers, based on known mean values for a healthy male population [42] and patients on hydrocortisone replacement therapy [18], at the 5% level of significance. A *p*-value of < 0.05 was considered statistically significant.

## Results

### Circulating serum cortisone/cortisol

**Fig. 1:**
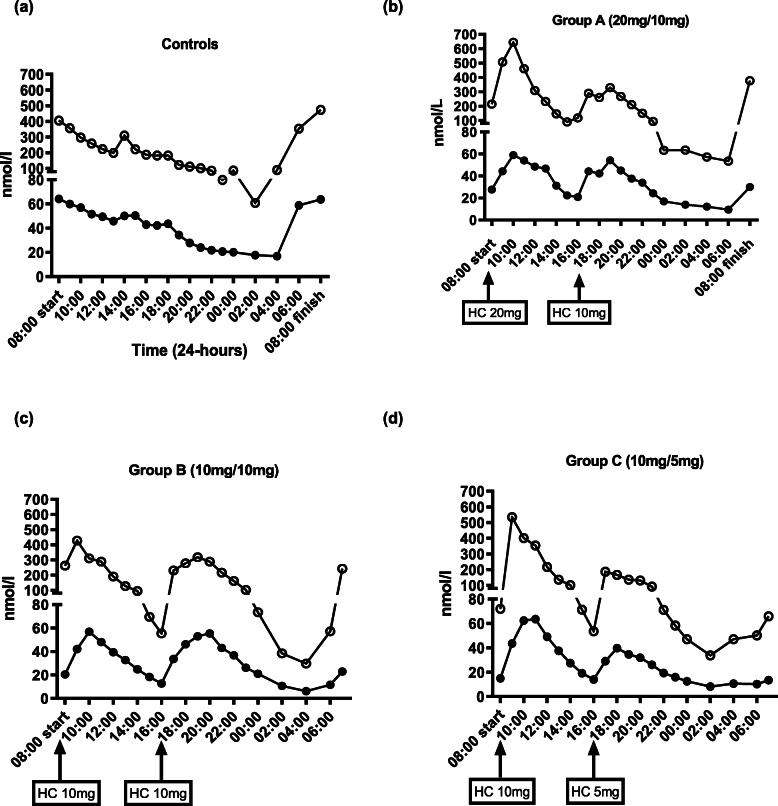
Mean 24-h serum total cortisol (open circles) and cortisone (closed circles) profile in (**a**) Controls (**b**) Group A (**c**) Group B (**d**) Group C. Hydrocortisone doses given at 08.00 h and 16.00 h

Circadian variations in serum cortisone and cortisol in healthy controls and study participants receiving the three different dose regimens of hydrocortisone are shown in Fig. 1. At 08.00 h, patients with adrenal insufficiency had significantly lower cortisol [median (IQR) 81 nmol/l (22–157) vs 391.5 nmol/l (326–488.5), *p* = 0.005] and cortisone [median (IQR) 20 nmol/l (5-32) vs 64.5 nmol/l (57.3–71.3), *p* < 0.0001] concentrations than healthy controls. Patients receiving hydrocortisone therapy had a higher AUC of cortisol [AUC median (IQR) 883.5 (709.5–1360) vs 616 (447.4–847.4), *p* = 0.019] and cortisone [AUC median (IQR) 216.5 (174.5–2475) vs 165.8 (132.4–196.4), *p* = 0.015] concentrations from 18.00 to 24:00, after taking their hydrocortisone at 16:00, compared to controls.

Fluctuations in serum cortisone concentrations in patients were found to parallel those for cortisol (albeit at lower concentrations) with peaks and troughs relating to the dosing schedule, Fig. 1. When data from all patients on hydrocortisone replacement therapy were analysed, we found a strong positive correlation between circulating serum cortisone and serum cortisol (*r* = 0.93, *p* = < 0.0001).

**Fig. 2:**
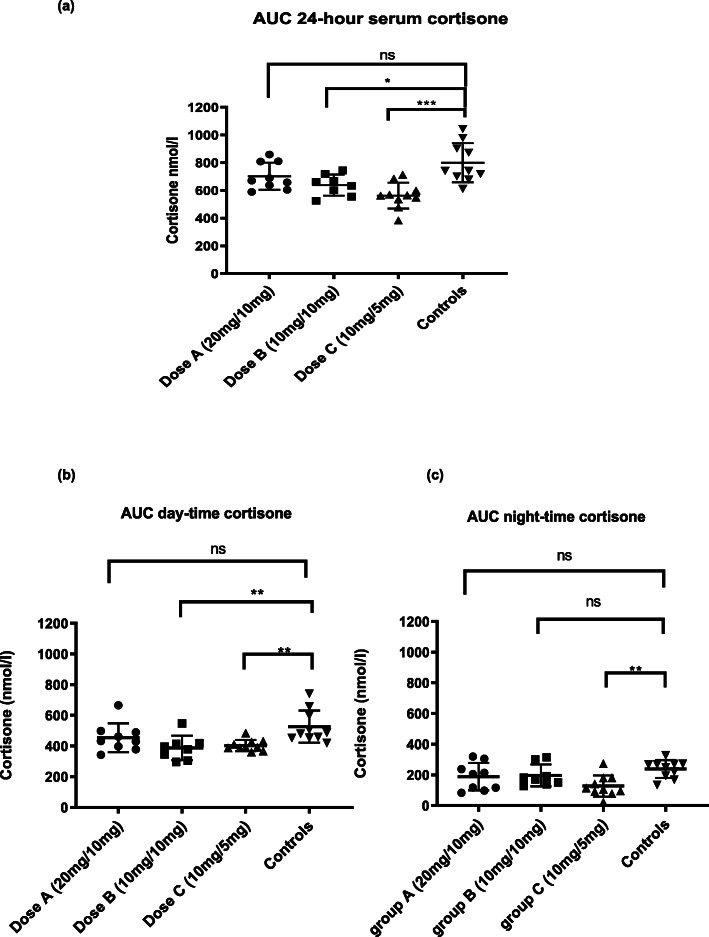
(**a**) Area under the curve (AUC) cumulative exposure of 24-h serum cortisone (**b)** AUC day-time cumulative exposure of cortisone **(c)** AUC night-time cumulative exposure of cortisone in patient groups and controls. ns, not significant, *p* value> 0.05, *p value < 0.05, **p value < 0.01, ***p value< 0.001

The area under the curve (AUC) of 24-h serum cortisone concentrations was significantly higher in patients on dose A (20 mg/10 mg) [670.5 (IQR 621–809.2)] compared to those on dose C (10 mg/5 mg) [562.8 (IQR 520.1–619.6), *p* = 0.01]. There was no significant difference in the AUC of 24-h serum cortisone concentrations between dose A (20 mg/10 mg) [670.5 (IQR 621–809.2)] and dose B (10 mg/10 mg) [647.8 nmol/L (IQR 566.9–706.3), *p* = 0.24] or between dose B (10 mg/10 mg) [647.8 nmol/L (IQR 566.9–706.3)] and dose C (10 mg/5 mg) [562.8 nmol/l (IQR 520.1–619.6), *p* = 0.09]. Patients on dose B and dose C had significantly lower 24-h serum cortisone concentrations than the healthy control group [AUC 742.3 (IQR 696.6–923.3)], *p* value = 0.01 and *p* = 0.0003, respectively. There was no significant difference in serum cortisone concentrations between patients on dose A (20/10mgs) compared to the control group (*p* = 0.10), Fig. 2.

### The relationships between serum cortisone/ cortisol and bone turnover markers

The concentrations of the bone turnover markers in the study population are detailed in Table 1.

**Table 1:** Concentrations of bone turnover markers in patients and controls. Data are expressed as mean (standard deviation) or median (interquartile ranges)

	Dose A (***n*** = 10) 20 mg/10 mg	Dose B (***n*** = 10) 10 mg/10 mg	Dose C (***n*** = 10) 10 mg/5 mg	Controls (***n*** = 10)
**PINP (μg/l)**	54.9 (36.4–139.5)	71.6 (42.9–126.7)	102.4 (54.9–166.1)	45.65 (37.8–30.2
**OC[1–49] (μg/l)**	21.9 (19.1–42.3)	26.3 (20.5–39.3)	34.2 (23.7–43.4)	18.6 (15.7–30.2)
**Bone ALP (μg/l)**	16.5 (10.6)	15.5 (7.9)	14.4 (7.6)	12.1 (3.5)
**CTX-I (μg/l)**	0.51 (0.29–0.96)	0.47 (0.26–0.64)	0.58 (0.29–0.84)	0.32 (0.27–0.56)
**TRACP5b (IU/l)**	2.92 (2.27–3.76)	2.69 (2.27–3.53)	2.76 (2.07–3.09)	2.79 (2.64–2.97)
**PINP:CTX-I ratio**	137 (43.5)	181 (79.3)	208 (56)	136 (49.2)

OC [1–49] = osteocalcin, *PINP* Procollagen type 1 peptide, *Bone ALP* Bone alkaline phosphatase, *CTX-I* C terminal cross-linking telopeptide, *TRACP5b* Tartrate resistant acid phosphatase 5b

#### Bone formation

**Fig. 3:**
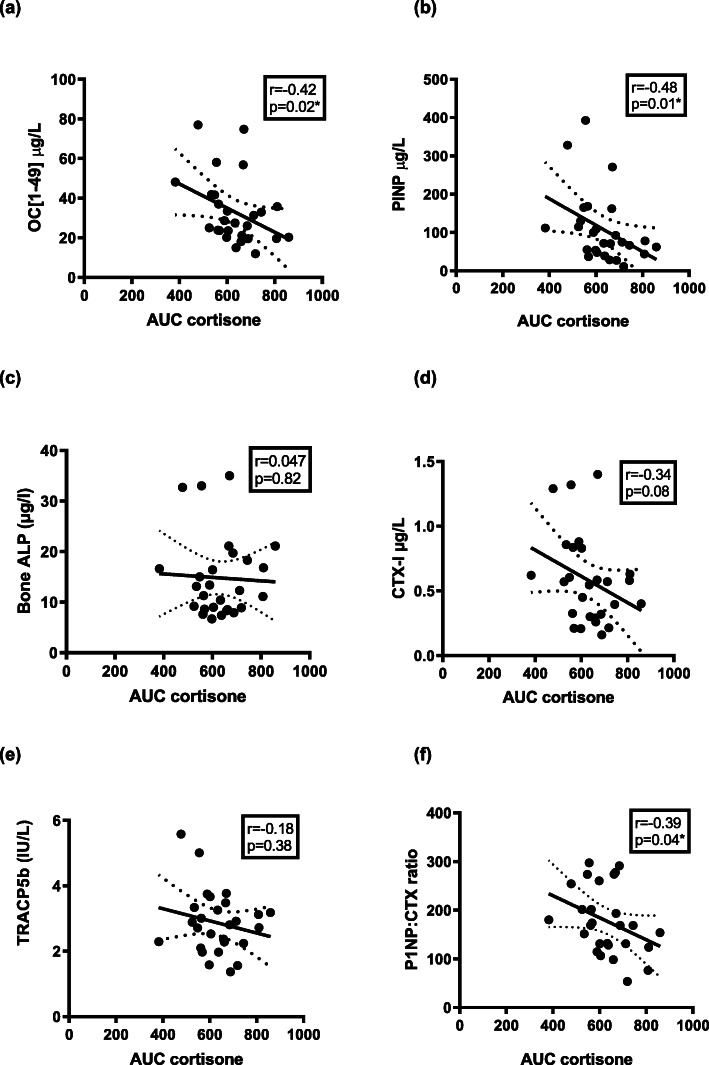
Correlation between circulating serum cortisone in all patients on hydrocortisone replacement with; Bone formation markers (**a**) OC [1–49], (**b**) PINP, (**c**) Bone ALP and Bone resorption markers (**d**) CTX-I, (**e**) TRACP5b and (**f**) PINP: CTX ratio. OC [1–49] = osteocalcin; PINP = procollagen type 1 peptide; Bone ALP = bone alkaline phosphatase; CTX-I = C terminal cross-linking telopeptide; TRACP5b = tartrate resistant acid phosphatase 5b

When all patients were combined, a significant negative correlation was observed between serum cortisone and bone formation markers, OC [1–49], [*r* = − 0.42, *p* = 0.03, Fig. 3a] and PINP [*r* = − 0.49, *p* = 0.01), Fig. 3b]. There was a negative correlation seen between serum cortisol and PINP (r = − 0.36, *p* = 0.07) but this did not reach significance, however, a significant negative correlation was shown between serum cortisol and OC [1–49], (*r* = − 0.57, *p* = 0.002), Table 2.

**Table 2:** Association of circulating serum cortisone (AUC) and total cortisol (AUC) with bone turnover markers in patients receiving hydrocortisone

All patients on HC Spearman Correlation (r)	Cortisone (AUC) ***r*** value, (***p*** value)	Cortisol (AUC) ***r*** value, (***p*** value)
**Bone Formation**		
**OC[1–49] (μg/l)**	−0.42, (*p* = 0.02)*	− 0.57, (*p* = 0.002)*
**PINP (μg/l)**	−0.49, (*p* = 0.01)*	−0.36, (*p* = 0.0689)
**Bone ALP (μg/l)**	0.05, (*p* = 0.82)	−0.06, (*p* = 0.78)
**Bone Resorption**		
**CTX-I (μg/l)**	−0.34, (*p* = 0.08)	−0.5, (*p* = 0.008)*
**TRACP5b (IU/l)**	− 0.18, (*p* = 0.38)	−0.2323, (*p* = 0.24)
**PINP: CTX-I ratio**	− 0.39, (*p* = 0.04)*	−0.4756, (*p* = 0.01)*

*HC* Hydrocortisone, *AUC* Area under curve, OC [1–49] = osteocalcin, *PINP* Procollagen type 1 peptide, *Bone ALP* Bone alkaline phosphatase, *CTX-I* C terminal cross-linking telopeptide, *TRACP5b* Tartrate resistant acid phosphatase 5b

*p < 0.05

To assess the relative importance of the diurnal rhythm of cortisone/cortisol, we assessed the diurnal variation of circulating serum cortisone/cortisol and the association with bone turnover markers, Table 3. There was a negative correlation between the AUC of night-time serum cortisone concentrations with the bone formation marker, OC [1–49] (*r* = − 0.41, *p* = 0.03). Similarly, there was a negative correlation between night-time serum cortisol with OC [1–49], however, this was less significant (*r* = − 0.36, *p* = 0.07). We also observed negative correlations between the AUC of night-time serum cortisone and PINP (*r* = − 0.34, *p* = 0.08) and serum cortisol with PINP (r = − 0.38, *p* = 0.05). There was a reciprocal relationship with the AUC of day-time trough cortisone levels and bone formation markers PINP (*r* = − 0.4, *p* = 0.03) and OC [1–49] (*r* = − 0.42, *p* = 0.03) in the patients receiving hydrocortisone, that was not observed in the control group.

**Table 3:** Association of circulating day-time and night-time serum cortisone (AUC) and total cortisol (AUC) with bone turnover markers in patients receiving hydrocortisone

All patients on HC Spearman Correlation (r)	Day-time Cortisone (AUC) ***r*** value, (***p*** value)	Day-time Cortisol (AUC) ***r*** value, (***p*** value)	Night-time Cortisone (AUC) ***r*** value, (***p*** value)	Night-time Cortisol (AUC) ***r*** value, (***p*** value)
**Bone Formation**				
**OC[1–49] (μg/l)**	−0.23, (*p* = 0.25)	−0.23, (*p* = 0.25)	−0.41, (*p* = 0.03)*	−0.36, (*p* = 0.07)
**PINP (μg/l)**	− 0.28, (*p* = 0.16)	−0.28, (*p* = 0.16)	−0.34, (*p* = 0.08)	−0.38, (*p* = 0.04)*
**Bone ALP (μg/l)**	− 0.08, (*p* = 0.7)	−0.0778, (*p* = 0.7)	− 0.03, (*p* = 0.9)	0.013, (*p* = 0.95)
**Bone Resorption**				
**CTX-I (μg/l)**	− 0.14, (*p* = 0.50)	−0.14, (*p* = 0.50)	−0.34, (*p* = 0.08)	−0.28, (*p* = 0.16)
**TRACP5b (IU/l)**	− 0.19, (*p* = 0.32)	−0.19, (*p* = 0.32)	0.021, (*p* = 0.92)	−0.12, (*p* = 0.55)
**PINP:CTX-I ratio**	− 0.28, (*p* = 0.14)	−0.29, (*p* = 0.14)	−0.12, (*p* = 0.55)	−0.29, (*p* = 0.13)

*HC* Hydrocortisone, *AUC* Area under curve, OC [1–49] = osteocalcin, *PINP* Procollagen type 1 peptide, *Bone ALP* Bone alkaline phosphatase, *CTX-I* C terminal cross-linking telopeptide, *TRACP5b* Tartrate resistant acid phosphatase 5b

*p < 0.05

#### Bone resorption

There was a negative correlation between AUC serum cortisol and the bone resorption marker CTX-I (*r* = − 0.5, *p* = 0.008), Table 2 which was not as strong between serum cortisone and CTX-I (r = − 0.34, *p* = 0.08), Fig. 3d. Both serum cortisone and serum cortisol negatively correlated with the bone-remodelling index, PINP: CTX-I ratio, with a stronger significance observed with serum cortisol (*r* = − 0.48, *p* = 0.012) than with serum cortisone (*r* = − 0.39, *p* = 0.04), Fig. 3f.

Night-time serum cortisone negatively correlated with CTX-I, but this was not significant (*r* = − 0.34, *p* = 0.08). We did not observe any significant correlations between day-time and night-time serum cortisone or cortisol with any other bone resorption markers, Table 3.

Due to the significant correlation between serum cortisone and serum cortisol and the small sample size we were not able to accurately adjust (using multiple regression analysis) to estimate the impact of each independent variable on bone turnover markers.

### Urinary cortisol metabolites and bone turnover markers

**Fig. 4:**
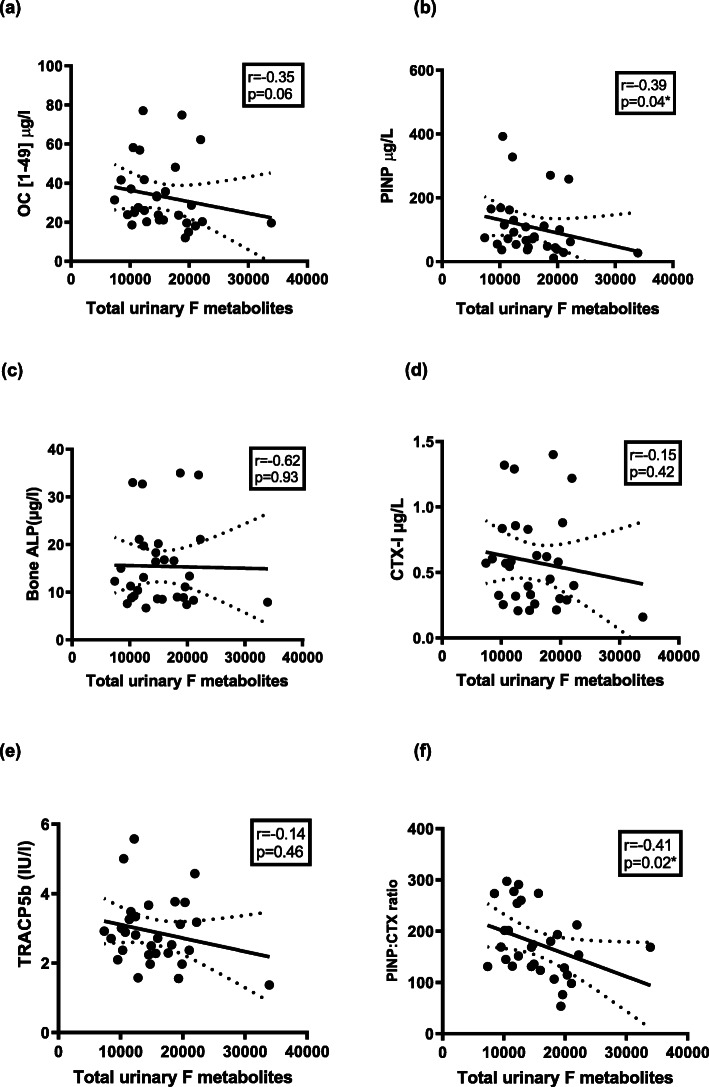
Correlation between total urinary cortisol metabolites in all patients on HC replacement with; Bone formation markers (**a**) OC [1–49], (**b**) PINP, (**c**) Bone ALP and Bone resorption markers (**d**) CTX-I, (**e**) TRACP5b, (**d**) PINP: CTX ratio. Total urinary F metabolites = Total urinary cortisol metabolites; OC [1–49] = osteocalcin; PINP = procollagen type 1 peptide; Bone ALP = bone alkaline phosphatase; CTX-I = C terminal cross-linking telopeptide; TRACP5b = tartrate resistant acid phosphatase 5b

Dose A (20 mg/10 mg) was associated with significantly higher total urinary cortisol (F) metabolites compared to the other dose regimens (*P* < 0.05 vs dose B, *P* < 0.001 vs dose C) and healthy controls (*P* < 0.01), while there was no difference between dose B (10 mg/10 mg), dose C (10 mg/5 mg) and control patients [35]. When combining the results of all patients receiving hydrocortisone replacement for analysis (but not controls), there was a negative correlation between total urinary cortisol metabolites with the bone formation marker, PINP (*r* = − 0.39, *p* = 0.04), Fig. 4b, and a trend towards significance with OC [1–49] (*r* = − 0.35, *p* = 0.06), Fig. 4a. There was a negative correlation between total urinary cortisol metabolites and the bone remodelling ratio, PINP: CTX-I ratio (r = − 0.41, *p* = 0.02), Fig. 4f.

As detailed in a previous publication [35], 11β-HSD1 activity was increased across all dose regimens compared to healthy controls and highest in dose A (20 mg/10 mg). When combining the results of all patients receiving hydrocortisone replacement for analysis (but not controls), the urinary THF + alloTHF/ THE ratio, a measure of global 11β-HSD1 activity did not correlate with any bone turnover markers. There was no difference in UFF/UFE, a marker of 11β-HSD2 activity, between the different HC doses and no observed correlation between UFF/UFE, with any bone turnover markers in the patient group.

The activities of the 5α and 5β-reductase enzymes can be inferred from measuring the ratio of 5α over 5β-reduced steroid metabolites, i.e. 5α-THF/THF and androsterone/etiocholanolone. There was a positive correlation between the androsterone/etiocholanolone ratio and the formation markers PINP (*r* = 0.35, *p* = 0.06) and OC [1–49] (*r* = 0.35, *p* = 0.06) and the bone-remodelling index PINP:CTX-I ratio (*r* = 0.37, *p* = 0.04). There were no significant correlations found with bone resorption markers or with 5α-THF/THF, Table 4.

**Table 4:** Association of urinary steroid metabolites with bone turnover markers in patients receiving hydrocortisone. Total urinary F metabolites = Total urinary cortisol metabolites; Andro = androsterone; Etio = etiocholanolone *p < 0.05

All patients on HC replacement (***n*** = 30) Urinary metabolites: r value (p value)	Total urinary F metabolites	THF + aTHF /THE	UFF/UFE	5a THF/THF	Andro/Etio
**Bone Formation**					
**OC[1–49] (μg/l)**	−0.35, (*p* = 0.06)	− 0.12, (*p* = 0.53)	0.09, (*p* = 0.62)	0.35, (*p* = 0.06)	0.5, (*p* = 0.004)*
**PINP (μg/l)**	−0.39, (*p* = 0.04)*	−0.18, (*p* = 0.32)	0.08, (*p* = 0.66)	0.35, (*p* = 0.06)	0.5, (*p* = 0.004)*
**Bone ALP (μg/l)**	−0.02, (*p* = 0.93)	−0.18, (*p* = 0.35)	0.29, (*p* = 0.11)	−0.07, (*p* = 0.7)	0.13, (*p* = 0.49)
**Bone Resorption**					
**CTX-I (μg/l)**	−0.15, (*p* = 0.42)	−0.18, (*p* = 0.35)	0.17, (*p* = 0.36)	0.17, (*p* = 0.38)	0.30, (*p* = 0.1)
**TRACP5b (IU/l)**	−0.14, (*p* = 0.46)	−0.31, (*p* = 0.09)	0.18, (*p* = 0.35)	−0.8, (*p* = 0.66)	0.18, (*p* = 0.33)
**PINP: CTX-I ratio**	−0.41, (*p* = 0.02)*	−0.15, (*p* = 0.45)	− 0.04, (*p* = 0.83)	0.37, (*p* = 0.04)*	0.58, (*p* = 0.008)*

## Discussion

We report that serum cortisol, cortisone and urinary total cortisol metabolites are associated with alterations in bone turnover markers in patients with adrenal insufficiency receiving commonly used doses of hydrocortisone replacement therapy. We also report that there is a dose-response relationship between serum cortisone and the dose of hydrocortisone and this impacts on markers of bone turnover in patients receiving hydrocortisone therapy. There is a greater impact of night-time cortisol and cortisone exposure than day-time exposure on bone turnover markers in patients receiving hydrocortisone replacement therapy.

We found the values of cortisone to accord well with previously published results for serum cortisone [43, 44]. Our study shows that serum cortisone fluctuates over the day in patients receiving hydrocortisone therapy, with the timing of peaks and troughs like those for cortisol. Cortisone excursions are also dependent on the dose of hydrocortisone ingested and are significantly different from those reported in healthy controls. While the overall concentrations of circulating cortisone may not be elevated compared to controls, we postulate that the fluctuations at a tissue level may have a potential impact on bone biology.

Previous studies have also reported cyclic variations in serum cortisone however, these studies used radioimmunoassay for cortisone measurement [43, 44]. Immunoassays have limited dynamic range particularly at lower concentrations and show cross-reactivity with structurally related metabolites. It has recently been recognized by the Endocrine Society that the performance of some immunoassays measuring cortisol and cortisone may be suboptimal for clinical use [45]. LC-MS provides a gold standard measure by which all routine assays are assessed [45]. Few studies have determined the simultaneous fluctuations and relationship of cortisol and cortisone by the gold standard method of LC-MS/MS [46]. It is important to highlight that most of serum cortisol is bound [80% bound to cortisol-binding globulin (CBG) and 10% to albumin] and is therefore of limited bioavailability. Serum cortisone binds with lower affinity to CBG and therefore may potentially lead to physiological glucocorticoid availability within tissues via conversion to cortisol by 11β-HSD1 [47, 48].

The process of bone remodelling is complex and targeted at multiple levels by glucocorticoids [33]. It is understood that glucocorticoids affect the function of multiple cell types, with the strongest evidence indicating osteoblasts as the main target [49]. The transcription of osteocalcin, an osteoblast-specific gene, is suppressed by glucocorticoids [50] and serum levels of osteocalcin are reduced in patients receiving glucocorticoids [51, 52]. Our study supports these findings, as we observed a significant negative correlation between serum cortisol and cortisone and OC [1–49] in our patients on HC replacement therapy.

There is limited data on the role of serum cortisone on bone physiology. In a cross-sectional study of healthy subjects (135 woman and 171 men), Cooper et al. found a negative correlation between serum cortisone and osteocalcin, which was stronger in men than women and independent of serum cortisol [21]. Interestingly, we found that night-time serum cortisone levels negatively correlated with bone formation markers, OC [1–49] and PINP, as did nocturnal serum cortisol with OC [1–49] and PINP but no significant correlations were seen between day-time serum cortisone or cortisol with any bone turnover markers. Bone turnover has a circadian rhythm in humans, with bone resorption and, to a lesser extent, bone formation increasing at night [53, 54]. Several studies have examined the role of cortisol in mediating the circadian rhythm of bone turnover, with conflicting results. Neilson et al. found that single oral doses of prednisolone (2.5 and 10 mg) given to healthy subjects inhibited and even reversed the nocturnal rise in serum osteocalcin levels [55]. Schlemmer et al. reported that hydrocortisone administered orally in divided doses to patients with adrenal insufficiency did not prevent the nocturnal increase in bone resorption [27]. Heshmati et al. inhibited endogenous cortisol synthesis using metyrapone and infused cortisol at either a variable rate (to mimic the physiological circadian variation in serum cortisol) or at a constant rate (to eliminate the cortisol rhythm) and assessed the circadian variation in bone formation and bone resorption under these two conditions [28]. They found that the morning rise in serum cortisol was responsible for the day-time nadir in serum osteocalcin levels and conversely the nocturnal increase in serum osteocalcin levels was a consequence of the declining evening and night-time cortisol levels. This suggests that nocturnal glucocorticoid exposure has potentially a greater influence on bone turnover, as was observed in our study population. We believe this has significant clinical implications with regard to the timing of glucocorticoid dosing, as patients (particularly those taking thrice-daily regimens) may be recommended to take their final hydrocortisone dose of the day in the late afternoon/ early evening and some patients with congenital adrenal hyperplasia have historically received glucocorticoids late at night to impact on the nocturnal rise in adrenal androgens in response to the nocturnal ACTH surge. If glucocorticoids are taken later in the day it may lead to higher levels of cortisol/ cortisone during night-time hours and thus have a greater negative impact on bone metabolism [56].

In our study, patients who were on hydrocortisone therapy had an increase in total urinary cortisol metabolites, which negatively correlated with bone formation markers PINP and OC [1–49]. This observation provides evidence that exogenous hydrocortisone is not simply excreted by the kidneys but is metabolized at a cellular level, leading to enhanced glucocorticoid tissue exposure and potentially deleterious effects on bone turnover. Tissue exposure to glucocorticoids is, in part, determined at the pre-receptor level; where 11β-hydroxysteroid enzymes play a central role. 11β-HSD1 is the predominant isozyme expressed in normal adult osteoblasts and osteoclasts, converting inactive cortisone to cortisol, and determines their exposure to active glucocorticoids. Cooper et al. previously observed that urinary measures of 11β-HSD1 activity (THF + 5αTHF/THE) predicted the reduction in bone formation markers, OC and PINP, in 20 healthy adult patients post oral prednisolone therapy (10 mg daily for 7 days) [57]. We have previously shown that in patients receiving oral HC replacement, there was an increase in 11β-HSD1 activity compared to the control group, however, in this study, we did not observe a significant correlation with urinary markers of 11β-HSD1 activity and bone formation markers [35]. This may reflect the lower glucocorticoid dose in our study population compared to the study by Cooper et al., and the fact that our patients were on stable hydrocortisone therapy for many years, compared to the study by Cooper et al. who measured the effect of a short exposure to high dose glucocorticoid therapy. The risk of bone loss tends to be highest in the acute phase post commencement of glucocorticoid therapy followed by a slower, steady-state of loss with chronic glucocorticoid use, as would have been the case in our patients.

In contrast to the enzyme 11β-HSD1, the A-ring reductases (5α-reductase and 5β-reductases) reduce local glucocorticoid availability by inactivating cortisol [58]. 5 alpha-reductase activities have been found in vitro in osteoblast-like cells [59]. We observed a positive correlation between 5α-reductase activity as measured by the urinary 5α THF/THF and ANDRO/ETIO ratios, with bone formation markers PINP and OC [1–49]. This would indicate that increased activity of 5-alpha reductases is associated with increased metabolism of active glucocorticoids to inactive glucocorticoids which is associated with an increase in bone formation. This may have implications for the bone health of patients who receive 5-alpha reductase inhibitors [60–63].

## Conclusion

In conclusion, changes in circulating cortisone and cortisol metabolites were associated with alterations in bone turnover. While further data is required, our data raises important questions regarding total daily dose, the impact of timing of glucocorticoid doses on bone health and the importance of bone-specific metabolism of glucocorticoids.

## Data Availability

The datasets used and/or analysed during the current study are available from the corresponding author on reasonable request.

## Abbreviations

ACTH: Adrenocorticotropin
AI: Adrenal insufficiency
ANDRO: Androsterone
ANOVA: Analysis of variance
AUC: Area under the curve
BMD: Bone mineral density
BMI: Body mass index
BTM: Bone turnover markers
bone ALP: Bone alkaline phosphatase
BP: Blood pressure
CTX-I: C terminal cross-linking telopeptide
E: Cortisone
ETIO: Etiocholanolone
F: Cortisol
GC: Glucocorticoid
GC-MS: Gas chromatography-mass spectrometry
HC: Hydrocortisone
HPA: Hypothalamic-pituitary-adrenal
IGF-1: Insulin-like growth factor I
IQR: Interquartile range
ITT: Insulin tolerance test
LC-MS: Liquid chromatography-mass spectrometry
LH: Luteinising hormone
OC: Osteocalcin
PINP: Procollagen type 1 peptide
PTH: Parathyroid hormone
QoL: Quality of life
Rpm: Revolutions per minute
T4: Thyroxine
TBG: Thyroid hormone-binding globulin
THE: Tetrahydrocortisone
THF: Tetrahydrocortisol
5a-THF: 5 alpha tetrahydrocortisol
TRACP5b: Tartrate resistant acid phosphatase 5b
TSH: Thyroid-stimulating hormone
UFE: Urinary free cortisone
UFF: Urinary free cortisol
WCM: Waist circumference
11β- HSD: 11 beta-hydroxysteroid dehydrogenase
25OH: 25 hydroxy
